# Targeting and Anchoring Tudor in the Pole Plasm of the *Drosophila* Oocyte

**DOI:** 10.1371/journal.pone.0014362

**Published:** 2010-12-15

**Authors:** Joël Anne

**Affiliations:** Department of Developmental Genetics, Deutsches Krebsforschungszentrum, Heidelberg, Germany; Katholieke Universiteit Leuven, Belgium

## Abstract

**Background:**

Germline formation is a highly regulated process in all organisms. In *Drosophila* embryos germ cells are specified by the pole plasm, a specialized cytoplasmic region containing polar granules. Components of these granules are also present in the perinuclear ring surrounding nurse cells, the nuage. Two such molecules are the Vasa and Tudor proteins. How Tudor localizes and is maintained in the pole plasm is, however, not known.

**Methodology/Principal Findings:**

Here, the process of Tudor localization in nuage and pole plasm was analyzed. The initial positioning of Tudor at the posterior pole of stage 9 oocytes was found to occur in the absence of a structurally detectable nuage. However, in mutants for genes encoding components of the nuage, including *vasa, aubergine, maelstrom,* and *krimper,* Tudor was detached from the posterior cortex in stage 10 oocytes, suggesting a prior passage in the nuage for its stability in the pole plasm. Further studies indicated that Valois, which was previously shown to bind *in vitro* to Tudor, mediates the localization of Tudor in the pole plasm by physically interacting with Oskar, the polar granule organizer. An association between Tudor and Vasa mediated by RNA was also detected in ovarian extracts.

**Conclusions/Significance:**

The present data challenge the view that the assembly of the polar granules occurs in a stepwise and hierarchical manner and, consequently, a revised model of polar granule assembly is proposed. In this model Oskar recruits two downstream components of the polar granules, Vasa and Tudor, independently from each other: Vasa directly interacts with Oskar while Valois mediates the recruitment of Tudor by interacting with Oskar and Tudor.

## Introduction

In many organisms, germline formation occurs in a specific region of the egg cytoplasm, the germ plasm. Ultrastructurally the germ plasm is composed of germinal granules and mitochondria [1], [2]. These granules are electron-dense structures acting as a repository for factors required in germline formation. In *Drosophila*, assembly of the germinal granules, or polar granules, requires the function of maternal-effect genes. Among these genes are *oskar (osk), vasa (vas)*, *tudor (tud)*, and *valois* (*vls*) which are essential for the formation of pole cells, the germline progenitors [3]. These genes produce proteins that localize to the polar granules [4]–[8]. The protein Osk acts by initiating granule formation during oogenesis and recruiting further components [9]. One of them corresponds to Vas which can directly interact with Osk [10]. Polar granule assembly is completed with the localization of proteins and various types of RNAs to the granules, including the mitochondrial ribosomal RNAs and *germ cell-less* transcripts [11], [12].

Three polar granule components, Tud, Vls, and Vas, are also present in a distinct structure at the periphery of nurse cell nuclei, the nuage [5], [8], [13]. Vas appears to be a pivotal organizer or nucleator of the nuage [14], as indicated by the absence of nuage in *vas* null ovaries [13]. Nuage components can be distributed in two types of structures, either as particles in cytoplasmic bodies surrounding germ cell nuclei, or as clusters assembled in a perinuclear ring [15]. Nuage particles are observed in the nurse cell cytoplasm, in ring canals connecting nurse cells to the oocyte, and to a lesser extent inside the ooplasm [16]. What are termed nuage particles are heterogeneous collections of related ribonucleoproteins (RNPs) that may differ in molecular composition and in their dynamics of assembly. Although nuage and polar granules form closely related structures, which suggest that components present in the nuage are precursors of those in the polar granules, the process leading to transport of components from the nuage to the polar granules appears complex [15]. The nuage can either act as a platform for the assembly of cytoplasmic RNPs or function as an intermediary step in this process. A prerequisite passage in the nuage for targeting components to the pole plasm is supported by the findings that all examined *vas* alleles affecting Vas localization in nuage also prevent Vas accumulation in pole plasm [13] and that Vas localization in pole plasm requires its association with Gustavus, a nuage component which only transiently accumulates in the pole plasm at the onset of vitellogenesis [17].

We previously showed that Tud localization in pole plasm requires *vls* function and that Tud and Vls can physically associate in vitro [8]. Furthermore, as shown by the absence of Tud in the pole plasm of embryos produced by females homozygous for the strong allele *vas^PD^* [5], [18], [19], Tud localization in pole plasm depends on the activity of Vas. The mechanism by which Tud become localized in the pole plasm remains however unknown. Recent observations suggested that the localization of Tud in the nuage is not required for its accumulation in the pole plasm [20].

Here the question of how Tud is targeted and anchored in the pole plasm is addressed, by analyzing Tud localization in nuage and pole plasm in mutants of genes encoding nuage-localized proteins and the physical relationship between Tud, Vls, and Vas. The present data suggest that events taking place in the nuage are necessary for correct assembly and stability of the polar granules in the oocyte and challenge the idea that the assembly of the polar granules occurs in a stepwise and hierarchical manner. A revised model for polar granule assembly is therefore proposed.

## Results

### Genetic control of Tudor localization in the nuage

**Figure 1:**
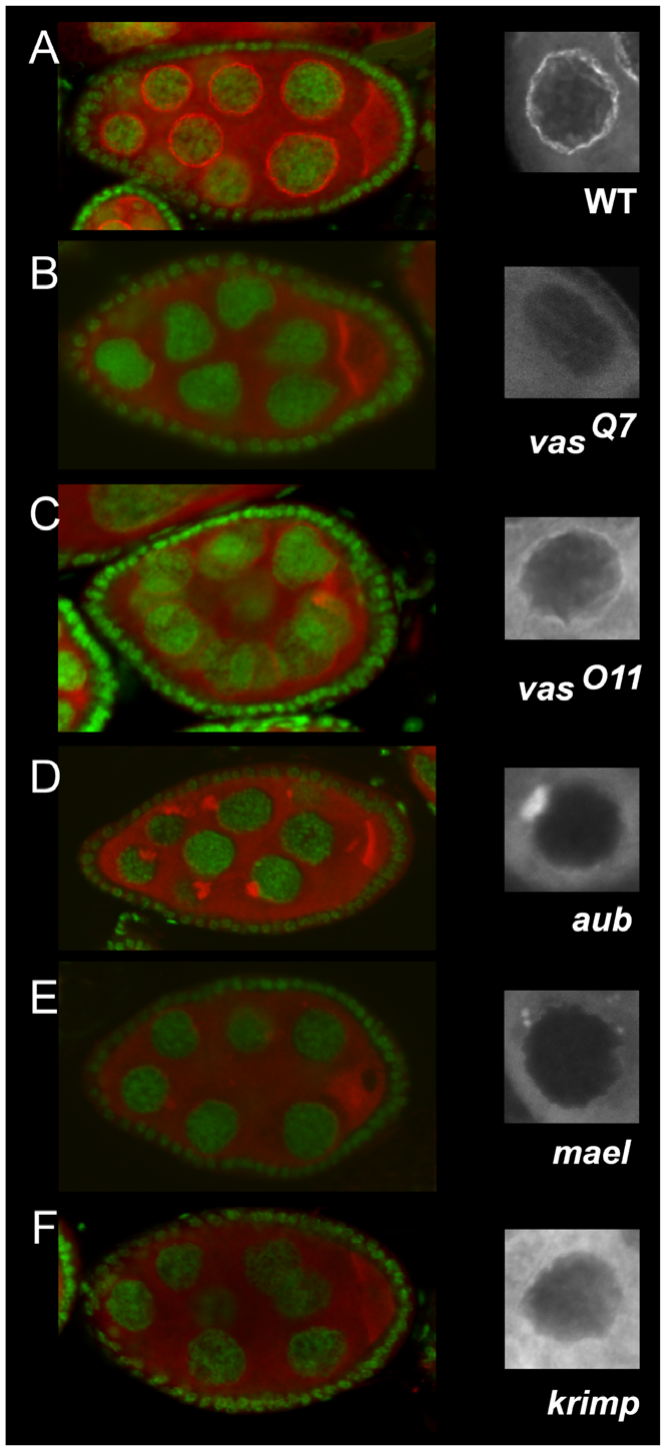
Genetic requirement for Tud localization in the nuage. Distribution of Tud and DNA in (A) wild-type, (B) *vas^Q7^*, (C) *vas^O11^*, (D) *aub^HN/N11^*, (E), *mael^M391^*/*Df(3L)79E-F*, and (F) *krimp^Qf065837^*/*Df(2R)Exel^6063^* stage 8 egg chambers. Tud (red), DNA (green). The right column displays a higher magnification of Tud distribution around wild-type and mutant nurse cell nuclei. (A) Tud normally accumulates at the periphery of the nurse cell nucleus. (B) Tud is absent from the nuage in the strong *vas^Q7^* allele and (C) is reduced in the weaker *vas^O11^* allele. (D) In *aub^HN/N11^* Tud aggregates in a single large spot bound to the nuclear membrane. (E) In *mael^M391^*/*Df(3L)79E-F* Tud accumulation in the nuage is severely impaired (left panel) but small aggregates containing Tud could be detected in the cytoplasm close to the nuclear membrane (right panel). (F) Tud localization in the vicinity of the nuclear membrane is drastically reduced in *krimp^Qf065837^*/*Df(2R)Exel^6063^* egg chambers.

To determine the genetic requirements for the localization of Tudor (Tud) in the nuage, egg chambers mutant for distinct components of the nuage, including Vasa (Vas) [19], Aubergine (Aub) [21], Maelström (Mael) [14], and Krimper (Krimp) [22], were examined. In wild-type egg chambers Tud formed a perinuclear ring around nurse cell nuclei (Fig. 1A). This localization was abolished in the strong allele *vas^Q7^* (Fig. 1B), whereas in the weaker *vas^011^* allele Tud perinuclear localization persisted, albeit to a lesser degree than in wild type (Fig. 1C). In the majority of *aub* stage 7 egg chambers Tud perinuclear localization was defective but in 32% of these egg chambers (n = 31) Tud was detected as a single large dot in direct contact with each nurse cell nucleus (Fig. 1D). This dot progressively disappeared and was never observed from stage 10 onward (data not shown). In *mael* egg chambers prior to stage 5 Tud formed first a detectable perinuclear ring around the nurse cell nuclei which disappeared progressively in later stages and left few residual dots at the nuclear periphery (Fig. 1E and data not shown), suggesting that *mael* may act in the maintenance of Tud in the nuage. In *krimp* egg chambers a significantly reduced level of Tud staining in the nuage was observed (Fig. 1F). The pathway leading to Tud accumulation in the nuage thus requires the activity of each of the four genes, which are therefore epistatic to *tud*.

### The activities of *vas, aub, mael,* and *krimp* are needed for Tudor stability in pole plasm

**Figure 2:**
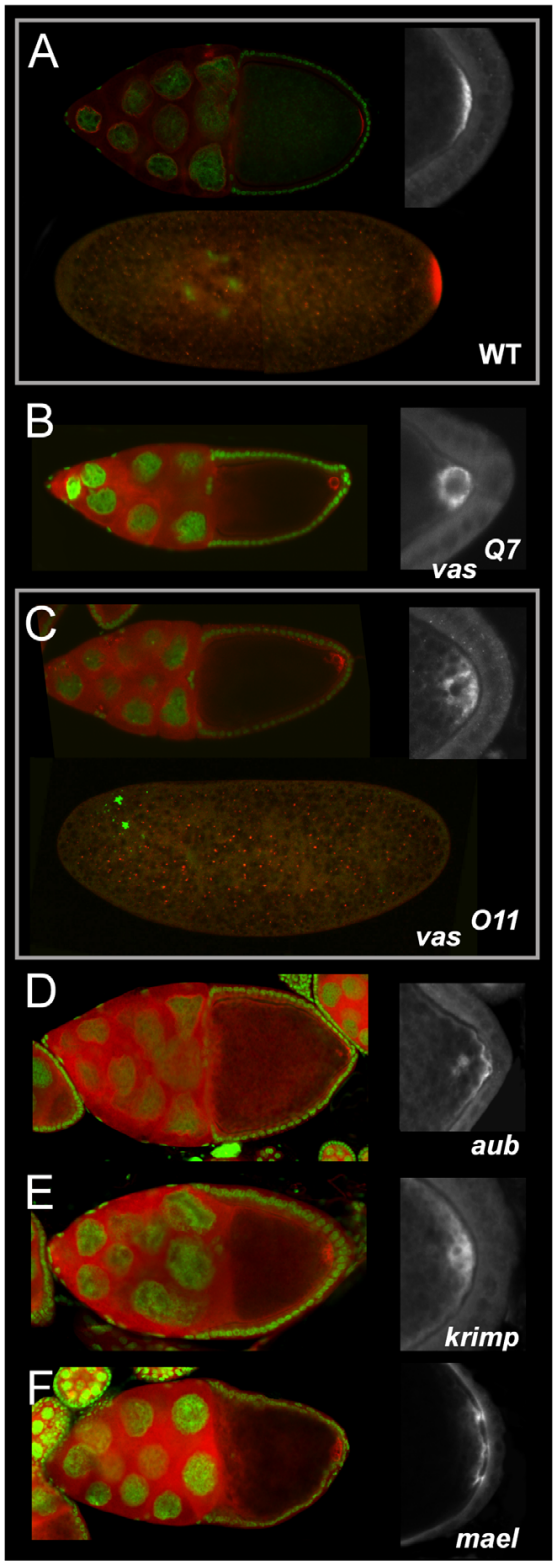
Components of the nuage are required for the stability of Tud in the pole plasm. Distribution of Tud in (A) wild-type, (B) *vas^Q7^*, (C) *vas^O11^*, (D) *aub^HN/N11^*, (E) *krimp^Qf065837^*/*Df (2R)Exel^6063^,* and (F) *mael^M391^*/*Df(3L)79E-F* stage 10 egg chambers and early cleavage embryos. Tud (red), DNA (green). Higher magnifications of the posterior cortex of the oocyte are provided on the right panels. (A, upper panel) In wild-type Tud forms a crescent at the posterior of the oocyte in stage 10 egg chamber and (lower panel) is maintained at the posterior pole during early embryogenesis. (B) In the strong *vas^Q7^* allele Tud accumulated at the posterior pole but became partially dissociated from the oocyte cortex and frequently formed a ring structure around hypothetical yolk particles. This structure stained negatively for lamin and thus did not correspond to the oocyte nucleus (data not shown). (C, upper panel) In the weaker *vas^O11^* allele Tud was poorly associated with the cortex and (lower panel) absent from the pole plasm during early embryogenesis. In (D) *aub^HN/N11^* and (E) *krimp^Qf065837^*/*Df(2R)Exel^6063^* stage 10 egg chambers Tud was associated with particles dispersed in the bulk cytoplasm. A fraction of the Tud particles formed a loose association with the cortex at the posterior pole. (F) In a *mael^M391^*/*Df(3L)79E-F* stage 10 egg chamber a weak crescent of Tud was associated with the posterior pole cortex through pillar structures.

To determine whether Tud localization in the nuage could be a prerequisite for its occurrence in the pole plasm, the distribution of Tud at the posterior pole of *vas, aub, mael,* and *krimp* oocytes was examined. As *vas* plays a pivotal role in nuage formation [14], the pole plasm localization of Tud in three *vas* alleles, *vas^PH165^* and *vas^Q7^* which produce no detectable protein [23], [24] and *vas^O11^* which makes a full-length protein but deficient in helicase activity [13] was analyzed. Surprisingly, despite a complete absence of Tud in the nuage, Tud was found to localize at the posterior pole in *vas^Q7^* oocytes of stage 9 egg chambers (Fig. S1). By contrast, in stage 10 *vas^Q7^* and *vas^PH165^* oocytes Tud is detached from the posterior cortex (Fig. 2B, upper panels, and Fig. S2, respectively). In *vas^Q7^* oocytes a ring of Tud stained-material around a spherical structure of unknown composition was observed in 23% of stage 10 egg chambers (n = 53). In the case of *vas^O11^* Tud is found in aggregated particles that were dispersed in the bulk cytoplasm of stage 10 egg chambers (Fig. 2C, upper panels). Tud signal could not be detected in 16% of stage 10 *vas^O11^* egg chambers (n = 62). This may result from defective localization of Tud in pole plasm or from a complete and early detachment from the posterior pole. The Tud particles remaining at the posterior cortex were unstable, as no Tud could be detected at the posterior pole in all *vas^011^* fertilized eggs (Fig. 2C, lower panel). The release of Tud from the posterior cortex which is observed in stage 10 *vas^011^* oocytes and completed in fertilized eggs indicates that Vas plays no docking role but contributes to Tud maintenance at this location, presumably through its helicase activity.

Examination of Tud distribution in *aub*, *mael*, and *krimp* oocytes revealed distinct patterns of localization in the pole plasm. In the majority of *aub*, *krimp* and *mael* stage 10 oocytes (Figs. 2D and E, and left panel of Fig.2F, respectively), particles containing Tud were observed in the cytoplasm. Tud material could not be detected in about 20% of stage 10 egg chambers mutant for either gene. In 17% of stage 10 *mael* egg chambers (n = 23) Tud was organized in two separated layers parallel to the cortex and partially joined by dense punctuated structures (right panel of Fig. 2F). This phenotype is specific for *mael* and was not observed in any other mutants.

The anchoring of Tud in pole plasm thus involves the activity of proteins structurally present in the nuage, but absent from the pole plasm, suggesting that localization of Tud in the nuage is required for its anchoring at the posterior pole.

### *In vitro* binding between Vls and Osk

**Figure 3:**
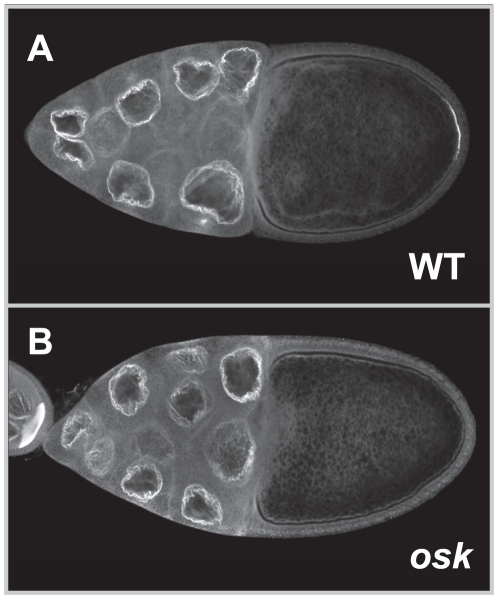
Recruitment of Tud by Osk protein. Distribution of Tud in (A) wild-type and (B) *osk^84^*/*Df(3R)pXT103* stage 10 egg chambers. In an *osk*-protein null egg chamber Tud localization in the pole plasm was abolished.

**Figure 4:**
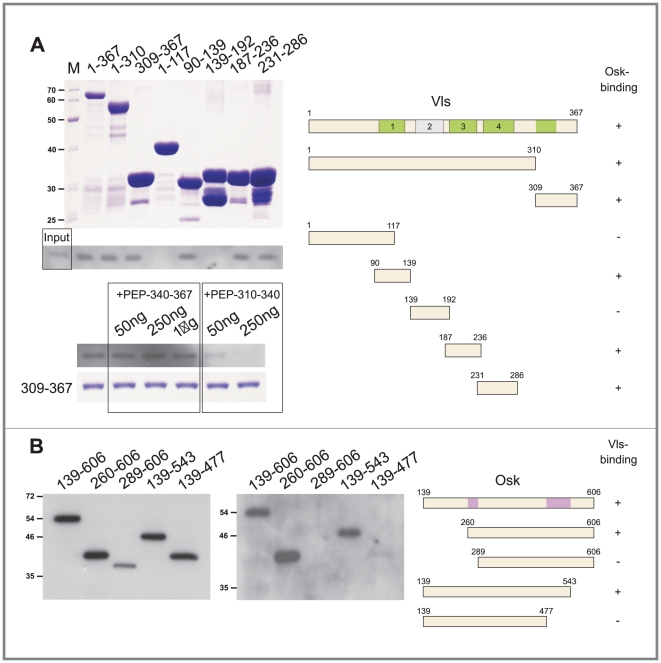
Vls interacts physically with the short Osk isoform. (A) Osk-binding domains in Vls. (Left, upper panel) Full length GST-Vls (367 amino acid residues) or derivatives were purified from bacterial extracts and the relative amounts of GST-fusion proteins were evaluated by SDS-PAGE followed by Coomassie staining. Amino acid numbers are given across the top. (Middle panel) The GST-fusion proteins were incubated with S•Tag-Osk. Bound S•Tag-Osk proteins were separated by SDS-PAGE electrophoresis and detected by immuno-blotting using alkaline phosphatase-conjugated S proteins. Input: one tenth of the protein extract was loaded on the gel. (Lower panel) To define more precisely the Osk-binding domain at the C-terminus of Vls, synthetic peptides corresponding to amino acid 310–340 and 340–367 of Vls were added to the binding assay containing the GST-Vls^309-367^ fusion fragment. The peptide 310-340 inhibited Osk binding to GST-Vls^309-367^. (Right) Representation of the GST-Vls fragments used for the mapping and summary of the results. The four WD-repeats are numbered and the putative Osk-binding domains of Vls are depicted in green. (B) Two internal regions in Osk are necessary for its binding to Vls. Full size S•Tag-short Osk isoform (447 amino acid residues) or derivatives were synthesized in vitro and incubated with full-size GST-Vls. Following separation by SDS-PAGE electrophoresis the bound S•Tag-Osk proteins were detected by immuno-blotting using alkaline phosphatase-conjugated S proteins. (Left panel) Input S•Tag-Osk proteins. (Middle panel) Bound S•Tag-Osk proteins. (Right) Representation of S•Tag-Osk constructs used for the mapping and summary of the results with the identified domains required for Vls-binding in Osk shown in pink.

Tud accumulates at the anterior margin of the oocyte following prepositioning of *osk* mRNA at this location [5]. However, the exact nature of the molecules recruiting Tud, either *osk* mRNA or Osk protein, remains unknown. Therefore whether *osk* mRNA was able to recruit Tud was first investigated by examining the localization of Tud in *osk* protein-null mutant ovaries. In these ovaries no Osk protein is synthesized although the *osk* mRNA is present at the posterior pole of the oocyte [6] and no Tud could be detected in the pole plasm (Fig. 3), indicating that Tud should be recruited by the Osk protein itself or by a component acting downstream of Osk. How the recruitment of Tud by Osk occurs was next asked. As a direct interaction between Tud and Osk is unlikely to occur [10], whether the Vls protein could mediate the recruitment of Tud by Osk was investigated. Vls was selected because previous analysis revealed that (1) Vls localizes to the pole plasm, (2) Vls can directly bind Tud *in vitro*, and (3) Tud is absent from the pole plasm of *vls* mutant oocytes [8]. To determine whether Vls physically interacts with Osk GST-pull down assays with either Vls or fragments of Vls fused to GST, and Osk or fragments of Osk fused to S•Tag [25] was performed. *In vitro* translated S•Tag-Osk polypeptides were incubated with immobilized GST-Vls proteins and, after washing, the bound S•Tag-Osk proteins were detected by using S-protein coupled to alkaline phosphatase. As shown in Figure 4A, Osk specifically bound immobilized full-length Vls as well as four discrete segments of Vls, including the first, third, and fourth WD repeat, and a C-terminal segment of Vls between amino acid residues 309 and 367. A synthetic peptide made of residues 310 to 340 of Vls was found to specifically inhibit the binding of S•Tag-Osk to the C-terminal segment of Vls, whereas no such inhibition was obtained with a Vls peptide corresponding to residues 340 to 367. Conversely, two regions of Osk (residues 260 to 289 and 477 to 543) necessary for the binding to Vls were identified (Fig. 4B). Osk and Vls can thus physically interact together, indicating that Vls may be the link between Osk and Tud in the pole plasm assembly pathway.

### RNA mediates the interaction between Vas and Tud

**Figure 5:**
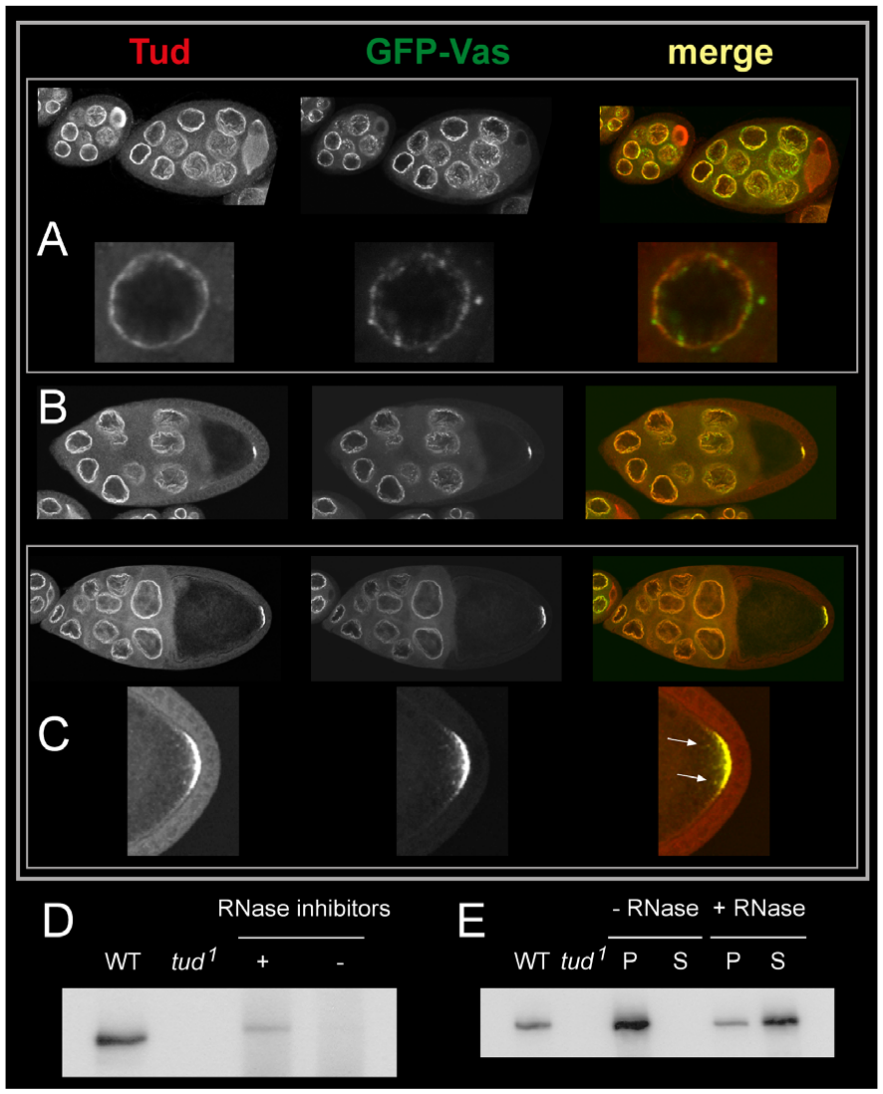
Relationship between Tud and Vas during oogenesis. (A-C) Distribution of GFP-Vas (green) and Tud (red) in wild-type (A) left, stage 6, and right, stage 8, (B) stage 9, and (C) stage 10 egg chambers. (A, upper panel) In stage 6 egg chambers Tud was enriched in the oocyte and in stage 8 transiently accumulated at the anterior margin of the oocyte, whereas GFP-Vas was predominantly detected in the nurse cells. (Lower panel) Both GFP-Vas and Tud decorated the membrane of nurse cell nuclei but the distribution of both proteins did not fully overlap; some Vas-containing particles were free of Tud. (B). In stage 9 egg chambers Tud and GFP-Vas began to accumulate at the posterior pole of the oocyte. (C, upper panel) Localization of GFP-Vas and Tud in nuage and pole plasm overlapped in a stage 10 egg chamber. (Lower panel) Higher magnification of the oocyte posterior cortex reveals the occurrence of particles containing both GFP-Vas and Tud in close proximity to the pole plasm (arrows). (D) RNA-dependent association of Tud with Vas. Immuno-detection of Tud in ovarian protein extracts separated by SDS-PAGE and blotted on PVDF membrane of wild-type females (first lane), homozygous *tud^1^* females (second lane), and affinity purified Vas-complexes isolated in presence (third lane) or absence of RNase inhibitors (fourth lane). Vas-complexes were purified from ovarian extracts representing ∼25-fold the amount of proteins loaded in the first lane. (E) RNase sensitive binding of Tud to Vas-complexes. Affinity purified Vas-complexes were isolated from ovarian protein extracts representing ∼75 fold the amount used in the first lane, as control, in presence of RNase inhibitors. Following purification the Vas-complexes were treated with RNase A and the Vas-complexes were isolated by centrifugation. Proteins in the pellet (P) and the supernatant (S) were separated and similarly analyzed for the occurrence of Tud as in D.

On the basis that *vas* activity contributes to the maintenance of Tud at the posterior pole of the oocyte the distribution pattern of Vas and Tud in growing egg chambers was first examined. For this purpose ovaries expressing a *GFP-vas* transgene were stained using anti-Tud antibodies. As previously reported, Tud accumulates in the oocyte of previtellogenic egg chambers [5], whereas GFP-Vas is only detected in the nurse cells [26]. In stage 7 egg chambers a transient accumulation of Tud at the anterior margin of the oocyte was detected, whereas Vas remained in the nurse cells (Fig. 5A, upper panels). By contrast, Tud and GFP-Vas were found to essentially co-localize in the nuage at distinct developmental stages (Fig. 5A–C) whereas Tud remained undetected in the cytoplasmic particles containing Vas (Fig. 5A, lower panels). The number of these particles decreased in stage 9 and 10 nurse cells. At stages 8-9 Tud was localized in the pole plasm where it overlapped with GFP-Vas (Fig. 5B). The pole plasm of all examined oocytes contained both molecules. No oocytes stained only for Tud or GFP-Vas could be detected. Interestingly, granules containing both GFP-Vas and Tud, which were located at distance from the cortex (arrow in Fig. 5C, lower panels) were detected. These granules were presumably in the process of being incorporated into the polar granules.

The constant co-localization of Tud and GFP-Vas in the oocyte suggests a physical interaction between both molecules. A possible link between Tud and Vas was investigated by isolated Vas-complexes from ovarian extracts of wild-type females with rabbit anti-Vas antibodies in presence or absence of RNase inhibitors. The components of the Vas-complexes were then separated by SDS-polyacrylamide gel electrophoresis and the presence of Tud was determined by Western blotting. Tud was specifically recovered in Vas-complexes when a cocktail of RNase inhibitors was added during extraction of the ovarian proteins (Fig. 5D). Osk protein could not be detected in the Vas-immunoprecipitates (data not shown), suggesting that these complexes occur in the bulk cytoplasm before they reach the posterior cortex. Furthermore a large proportion of the Tud protein could be released from the Vas-complex by treatment with RNase (Fig. 5E). These results indicate that the interaction between Tud and Vas is mediated by RNA molecules, whose nature remains to be determined.

### Mapping of Osk-binding domain in Vas

**Figure 6:**
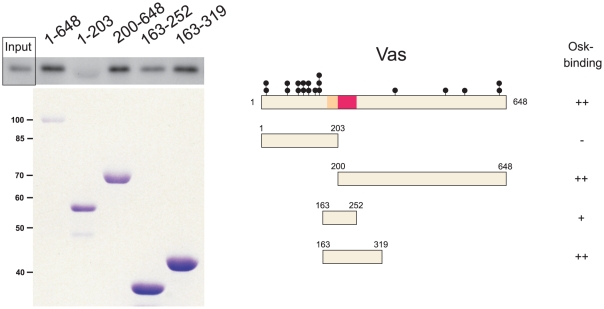
Delimitation of the Osk-binding domain in Vas. Full length GST-Vas (648 amino acid residues) or derivatives were purified from bacterial extracts and incubated with S•Tag-Osk. (Left, upper panels) Following separation by SDS-PAGE the bound S•Tag-Osk proteins were detected by immuno-blotting using alkaline phosphatase-conjugated S proteins. Input: one tenth of protein extract was loaded on the gel. (Lower panel) The amount of GST-Vas proteins was visualized by Coomassie staining. (Right) Representation of the GST-Vas fragments used for mapping and summary of the results. The RG repeats are indicated by black circles and the Gustavus- and Osk-binding domains of Vas are depicted as yellow and red boxes, respectively.

As Vas and Osk interacts in the yeast two-hybrid system [10], a GST-pull down assay was used to determine more precisely which region of Vas can physically interact with Osk. For this purpose Vas or fragments of Vas were fused to GST, and Osk to S•Tag and in vitro translated S•Tag-Osk polypeptides were incubated with immobilized GST-Vas proteins. The bound S•Tag-Osk proteins were then detected by using alkaline phosphatase coupled to S-protein. A region internally located after the N-terminal RG-rich region of Vas and Gustavus-binding domain [17] was found to mediate this interaction (Fig. 6). This result suggests that a region distinct from the RG-rich domain is involved in the association between Osk and Vas.

## Discussion

To gain insights into the mechanism of protein localization in *Drosophila* egg chambers the process by which Tud becomes incorporated into two related germline specific structures, the nuage and the polar granules, was analyzed.

### Assembly of Tud in the nuage

Attempts have been made to establish a hierarchical relationship among known components of the nuage [14], [22]. These analyses have revealed a complex pattern of dependence in which Vas plays a critical role in the recruitment of Aub, Mael, and Krimp, although Vas occurrence in the nuage may partially depend on proper Aub localization [22]. In addition Mael localization in the nuage may require Aub and Krimp [14], [22]. The present study showed that Tud perinuclear accumulation failed in *vas^Q7^*, a null *vas* allele, underlining the critical function of Vas, and was altered in *aub, mael,* and *krimp* mutants, indicating that Tud may act downstream of all examined factors in the pathway leading to nuage formation. The possibility that Tud is normally targeting to the nuage, but the nuage itself is delocalized in these mutants can be ruled out by the observation that Vasa and Aubergine are correctly localized in the nuage of *krimp* mutant egg chambers [22].

Interestingly in *aub* mutants the localization of Tud in the nuage is reminiscent, albeit not similar to that of Krimp, which forms aggregates in nurse cell cytoplasm [22]. By comparison to Krimp aggregates, which are found as numerous punctuate structures frequently observed at the proximity of nuclei, Tud aggregates in *aub* nurse cells were larger in size and less numerous. By contrast, in *mael* egg chambers Tud aggregates were of small size and only detected from stage 5 onwards in the vicinity of nurse cell nuclei. The variety of Tud structures seen in the different mutants suggests a complex and dynamic mechanism in nuage assembly involving multiple interactions.

### Relationship between nuage and polar granules

Two distinct roles have been assigned to the nuage. On the one hand the nuage may contribute to the formation of RNP complexes [15], and, on the other hand, it may repress the expression of specific selfish genetic elements, by producing repeat-associated small interfering RNAs [22].

The present study suggests that additional events may take place in the nuage in order to stabilize Tud at the posterior pole of the oocyte. As Krimp is a protein present in perinuclear foci, but not in the pole plasm, analysis of the localization of Tud at the posterior pole of *krimp* stage 10 oocyte suggested that the stability of Tud at this location depends on the activity of Krimp in nuage. Pole plasm instability of Tud in the different mutants so far tested is quite similar, with the exception of *mael*. In *vas*, *aub*, and *krimp* mutant oocytes, a marked proportion of Tud-containing particles are dispersed in the ooplasm. In contrast, in *mael* oocytes the pole plasm crescent seems to be detached from the cortex as a compact structure, with only few “pillars” connecting the crescent to the cortex. The detachment of the polar granules could be explained by the pleiotropic nature of *mael*; in particular, *mael* is required for early mRNA localization within the oocyte and premature cytoplasmic motion is observed in stage 8 *mael* mutant oocytes [27]. Although a role of nuage-localized proteins in cytoplasmic particles cannot be excluded, these data suggest that the nuage plays a critical role in the maintenance of Tud at the posterior pole but is dispensable for the transport of Tud to this location.

### Pole plasm assembly

The core protein components of the polar granules consist of Osk, Vas, and Tud. Osk is the pivotal organizer which anchors other granule components and controls granule morphology [9], [28]. Based mainly on the normal localization of Vas in *tud* embryos and the defective localization of Tud in *vas* embryos [18], [19], a pathway for pole plasm assembly has been proposed in which *tud* acts downstream of *vas* (see, for example, [29]). The present analysis of Tud protein localization in *vas* mutant egg chambers challenges this conclusion by showing that the assembly of the pole plasm does not occur in a stepwise fashion in which the localization of one protein depends on the localization of the preceding one. In particular, Tud localizes at the posterior cortex in stage 9 *vas* oocytes. Tud could thus be targeted to the pole plasm independently of Vas.

By which mechanism become Vas and Tud incorporated in the pole plasm? Although both Tud and Vas are directly localized in the pole plasm as proteins translated from mRNAs present in the nurse cells [5], [18], [19], each protein reaches the posterior pole of the oocyte through distinct ways. Vas is directly transported to the pole plasm whereas Tud displays a transient anterior localization before its association with the posterior pole [5]. A mechanism involving a passage of Vas through the nuage has been assumed on the basis that all known *vas* mutations affecting perinuclear accumulation of Vas also entail its posterior pole localization [13]. Furthermore, Vas binding to Gustavus (Gus) in the nuage is required for its incorporation into the pole plasm [17]. Similar to Vas, Gus forms ribonucleoprotein particles accumulating at the periphery of nurse cell nuclei constituting perinuclear nuage clusters. A mutated form of Vas lacking the Gus-binding site is still able to associate with the periphery of nurse cell nuclei but is absent from the oocyte posterior pole [17], suggesting that in order to access to pole plasm Vas should be modified or bound to a “cargo” in the perinuclear region of the nurse cells.

**Figure 7:**
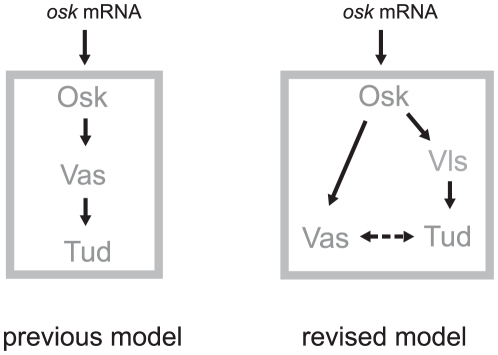
Scheme of polar granule assembly. This model is mainly based on the finding that Tud and Vas can localize in polar granule independently from each other and that Vls mediates the localization of Tud by binding to both Tud and Osk. An association between Vas and Tud could be mediated by RNA or through protein-protein interaction (See Discussion).

Tud localization in the pole plasm was found to be synchronous with that of Vas and similarly relies on Osk synthesis. In contrast to Vas binding to Osk, no physical interaction could be detected in the yeast-two hybrid system between Osk and JOZ, 9A1 or 3ZS+L Tud fragments [10], suggesting that Tud recruitment by Osk is different from that of Vas. Our previous work shows physical interaction between Vls and Tud [8] and the present work indicates that Vls could similarly interact with Osk. Based on this finding it is possible to envisage that Vls, a component of the pole plasm [8], forms a link between Osk and Tud. Accordingly, Tud localization in pole plasm was abolished in *vls* mutant egg chambers [8]. Furthermore, the present work showed that Tud could reach the pole plasm in absence of *vas* activity. Collectively, these data led me to propose a revised model for polar granule assembly (Fig. 7) in which two different pathways direct the localization of Vas and Tud in the polar granules. Osk may directly bind Vas whereas its interaction to Tud is mediated by Vls.

In addition, the process of pole plasm assembly may involve an interaction between Vas and Tud. This interaction could stabilize the structure containing both proteins. Two mechanisms can be envisaged for stabilizing Vas and Tud in polar granules. The first mechanism may involve an RNA-mediated association, as supported by the finding that Vas interacts with Tud through RNA. In a second mechanism, Vas and Tud may bind together through a weak association of the methylated RG domains of Vas and one or several Tudor domains of Tud. As previously reported SmB can bind to Tudor domains of Tud when the arginine residues in its RG-repeats are methylated [30]. As Vas contains a high number of clustered RG repeats whose arginine residues can be potentially methylated [31], it is possible to envisage that methylated forms of Vas could bind to one or more of the multiple Tudor domains. Interestingly the RG-rich domain of Vas is not required for interaction with Osk, leaving the possibility that this domain could mediate interaction with other proteins, such as Tud. This possibility could be tested experimentally when molecular events taking place within the polar granules would be accessible to biochemical analysis.

## Materials and Methods

### Fly strains

The wild-type fly strain and the recipient stock for P element transformation used in this study were Oregon R and *w^1118^*, respectively. The mutant alleles and allelic combinations were *b vas^Q7^ pr*/*CyO*, *b vas^O11^ pr cn*/*CyO* [32], *vas^PH165^*/*CyO* [23], *tud^1^*/*CyO*, *tud^1^*/*tud^WE8^* [33], *mael^M391^*/*Df(3L)79E-F* [27], *aub^HN/N11^* [34], *krimp^f06583^*/*Df(2R)Exel^6063^* [22], *osk^84^*/*Df(3R)pXT103* [35]. The *P_vas_-GFP-Vas* line [26] was given by A. Nakamura. Flies were grown at 25°C on corn/agar medium. Dry yeast was added to the medium the day before females were dissected for ovary preparation.

### Molecular Biology

Plasmid constructs were generated by amplification of the desired fragments by PCR (High Fidelity PCR Master; Roche), and were subcloned into appropriate vectors. *osk* and *vas* cDNA plasmids were kindly provided by A. Ephrussi and P. Lasko, respectively.

### Immuno-precipitation

Fly ovaries were dissected with forceps, collected in 1xPBS buffer in a microcentrifuge tube, and centrifuged at 10,000xg for one minute at 4°C. The supernatant was removed and the ovaries were suspended and homogenized in 125 µl of ice-cold IP buffer (145 mM NaCl, 10% glycerol, 1 mM MgCl_2_, 1.5 mM NaH_2_PO_4_, 8 mM Na_2_HPO_4_ [pH 7.4], 0.5% NP-40) containing protease inhibitors (complete EDTA-free protease inhibitor cocktail; Roche). Lysate was clarified by centrifugation at 14,000xg for 10 min at 4°C. The supernatant was removed and mixed with anti-Vas antibodies coupled to protein-A beads (90 units of RNasin were eventually added) for 5 hours at 4°C on a rotator. The protein-A beads were separated at low speed centrifugation, suspended in IP buffer, and washed 5 times. Proteins associated with the beads were then analyzed on a western blot for the presence of Tud by using an enhanced chemiluminiscence system. For the release experiment the immuno-complexes were washed extensively and incubated for 20 minutes at room temperature with 50μg of RNase A (Roche).

### Immunocytochemistry

Ovaries were dissected in PBS, fixed in 4% paraformaldehyde:heptane (1∶1) in PBS for 12 minutes, washed four times for 20 min in 0.1% Tween 20 in PBS (PBT), treated one hour in 1% Triton X-100 in PBS, and blocked for 1 h in 2% BSA in PBT. Primary antibody incubation was performed in 0.5% BSA, 5% normal goat serum in PBT overnight at 4(C, followed by several washes in PBT. Egg chambers were mounted in Elvanol. GFP-Vas egg chambers were fixed in 4% paraformaldehyde in PBS for 10 minutes, washed four times for 10 min in PBT, immunostained as described above, and mounted in Glycerol:PBS, 1∶1. Confocal data were acquired as single images with a Zeiss LSM 510 or Nikon Ellipse microscope. Primary antibodies were rabbit anti-Tud (TUD65) made against the C-terminal region of Tud protein (residues 2189–2515) [36] and rabbit anti-Osk antibodies [6]. Specificity of the anti-Tud serum was controlled by immunostaining *tud* mutant egg chambers. Only background signal could be detected at all stages (Fig. S3). Chromatin was visualized by staining with Oli-Green (Molecular Probes). Cy3-conjugated secondary antibodies (Jackson ImmunoResearch Laboratories) were used at 1∶200.

### GST Pull-Down Assay

Full-length coding sequence of the *vls* and *vas* cDNAs were subcloned into pGEX6P2 (GE Healthcare) and the fragment of *osk* cDNA corresponding to the short Osk isoform into pCITE-4 (Novagen). Recombinant proteins were synthesized in vitro using the TNT T7 Coupled Reticulocyte Lysate System (Promega) in the presence of unlabeled amino acids. GST-fusion proteins expressed in *E. coli* were purified with glutathione sepharose (GE Healthcare) and washed with binding buffer (20 mM HEPES, [pH 7.8], 10% glycerol, 300 mM NaCl, 0.1% sodium deoxycholate, 0.1% NP40, and 0.1% Triton X-100) plus protease inhibitors (Complete EDTA free from Roche; 1∶50 dilution). Recombinant proteins (10% of the reaction volume) were added to this mixture (in 1 ml) and incubated for 3 hr at room temperature. The beads were washed six times (10 minutes each) with binding buffer, boiled in 2xSDS loading buffer, and the proteins were separated by electrophoresis on 10% SDS-polyacrylamide gels. After transfer to a polyvinylidene difluoride (PVDF) membrane, the bound proteins were detected by Western blotting using an S-protein Alkaline Phosphatase conjugate (Novagen).

## Supporting Information

Figure S1 Tud accumulates in the pole plasm in stage 9 *vas* oocytes. Distribution of Tud in a *vas^Q7^/Df(2L)A72* stage 9 egg chamber.
(1.60 MB EPS)

Figure S2 *vas* activity is required for the anchoring of Tud in the pole plasm. Distribution of Tud in a *vas^PH165^/Df(2L)A72* stage 10 egg chamber. High magnification of the posterior cortex of the oocyte is provided.
(7.44 MB EPS)

Figure S3 Anti-Tud antibodies are specific for Tud. Distribution of Tud in (top) *tud^1^*/CyO and (bottom) *tud^1^* ovaries. Previtellogenic and stage 9-10 egg chambers were stained with anti-Tud antibodies. In *tud* mutant egg chambers only background signal could be detected. No specific staining in either pole plasm or nuage is observed. Samples were processed in parallel and same confocal settings were used for image acquisition.
(4.42 MB EPS)
